# Perception and Awareness of Diabetes Risk and Reported Risk-Reducing Behaviors in Adolescents

**DOI:** 10.1001/jamanetworkopen.2023.11466

**Published:** 2023-05-03

**Authors:** Patricia Chu, Arya Patel, Vicki Helgeson, Andrea B. Goldschmidt, Mary Katherine Ray, Mary Ellen Vajravelu

**Affiliations:** 1 Division of Endocrinology & Diabetes, Children’s Hospital of Philadelphia, Philadelphia, Pennsylvania; 2 Division of Endocrinology, Diabetes and Metabolism, University of Pennsylvania, Philadelphia; 3 University of Pittsburgh, Pittsburgh, Pennsylvania; 4 Department of Psychology, Carnegie Mellon University, Pittsburgh, Pennsylvania; 5 Department of Psychiatry, University of Pittsburgh, Pittsburgh, Pennsylvania; 6 Department of Psychiatry, Washington University in St Louis School of Medicine, St Louis, Missouri; 7 Division of Pediatric Endocrinology, Diabetes, and Metabolism, UPMC Children’s Hospital of Pittsburgh, Pittsburgh, Pennsylvania; 8 University of Pittsburgh School of Medicine, Pittsburgh, Pennsylvania

## Abstract

**Importance:**

Lifestyle change is central to diabetes risk reduction in youth with overweight or obesity. Feeling susceptible to a health threat can be motivational in adults.

**Objective:**

To evaluate associations between diabetes risk perception and/or awareness and health behaviors in youth.

**Design, Setting, and Participants:**

This cross-sectional study analyzed data from the US National Health and Nutrition Examination Survey 2011 to 2018. Participants included youths aged 12 to 17 years with body mass index (BMI) in the 85th percentile or higher without known diabetes. Analyses were conducted from February 2022 to February 2023.

**Main Outcomes and Measures:**

Outcomes included physical activity, screen time, and attempted weight loss. Confounders included age, sex, race and ethnicity, and objective diabetes risk (BMI, hemoglobin A_1c_ [HbA_1c_]).

**Exposures:**

Independent variables included diabetes risk perception (feeling at risk) and awareness (told by clinician), as well as potential barriers (eg, food insecurity, household size, insurance).

**Results:**

The sample included 1341 individuals representing 8 716 794 US youths aged 12 to 17 years with BMI in the 85th percentile or higher for age and sex. The mean age was 15.0 years (95% CI, 14.9-15.2 years) and mean BMI *z* score was 1.76 (95% CI 1.73-1.79). Elevated HbA_1c_ was present in 8.6% (HbA_1c_ 5.7%-6.4%: 8.3% [95% CI, 6.5%-10.5%]; HbA_1c_ ≥6.5%: 0.3% [95% CI, 0.1%-0.7%]). Nearly one-third of youth with elevated HbA_1c_ reported risk perception (30.1% [95% CI, 23.1%-38.1%), while one-quarter (26.5% [95% CI, 20.0%-34.2%]) had risk awareness. Risk perception was associated with increased TV watching (β = 0.3 hours per day [95% CI, 0.2-0.5 hours per day]) and approximately 1 less day per week with at least 60 minutes of physical activity (β = −1.2 [95% CI, −2.0 to −0.4) but not with nutrition or weight loss attempts. Awareness was not associated with health behaviors. Potential barriers had mixed associations: larger households (≥5 members vs 1-2) reported lower consumption of non–home-prepared meals (OR 0.4 [95% CI, 0.2-0.7]) and lower screen time (β = −1.1 hours per day [95% CI, −2.0 to −0.3 hours per day), while public insurance (vs private) was associated with approximately 20 fewer minutes per day of physical activity (β = −20.7 minutes per day [95% CI, 35.5 to −5.8 minutes per day]).

**Conclusions and Relevance:**

In this cross-sectional study including a US-representative sample of adolescents with overweight or obesity, diabetes risk perception and awareness were not associated with greater engagement in risk-reducing behaviors in youth. These findings suggest the need to address barriers to engagement in lifestyle change, including economic disadvantage.

## Key Points

**Question:**

Among youths at higher risk for type 2 diabetes due to overweight or obesity, are perception and awareness of diabetes risk associated with risk-reducing health behaviors?

**Findings:**

In this cross-sectional study of 1341 individuals representing 8 716 794 US youth with body mass index in the 85th percentile or higher, greater perception of diabetes risk was associated with lower physical activity and higher screen time, whereas awareness was not associated with health behaviors. Barriers, including measures of economic disadvantage, were associated with lower physical activity.

**Meaning:**

These findings suggest the need to address barriers to engagement in risk-reducing behaviors beyond risk perception and awareness, including economic disadvantage.

## Teaser

This cross-sectional study examines whether there are associations between diabetes risk perception and/or awareness and health behaviors in youth.

## Introduction

Prediabetes has more than doubled in prevalence among adolescents in the past 2 decades, now affecting approximately 40% of youth with obesity.^1,2^ Youth-onset type 2 diabetes, also increasing in incidence,^3^ leads to microvascular complications within the first 2 decades after diagnosis in approximately 80% of individuals.^4^ Because no medications have demonstrated reduction in progression from prediabetes to type 2 diabetes in youth,^5^ the primary treatment remains intensive lifestyle changes.^6^ Unfortunately, such change remains a substantial challenge.

As posited by the Health Belief Model, an individual’s engagement in a health-promoting behavior is driven by: (1) disease risk perception; (2) barriers to and benefits of change; and (3) cue to act, such as a new awareness of a diagnosis.^7^ In a study of adults with prediabetes, those who were aware of their diagnosis were more likely to report engaging in risk-reducing behaviors including nutrition or physical activity changes,^8^ although other studies have been mixed.^9,10^ Due to developmental differences, adolescents perceive and respond to health risks differently than adults.^11^ If, among adolescents, diabetes risk perception and awareness are linked to positive health behavior change, this could serve as further justification to recognize and diagnose prediabetes in youth.^12^

In this study using nationally representative pediatric data, we investigated whether diabetes risk perception, risk awareness, and potential barriers to behavior change were associated with diabetes risk-reducing health behaviors (ie, physical activity, nutrition) among adolescents at higher risk for diabetes based on elevated body mass index (BMI, calculated as weight in kilograms divided by height in meters squared) in the 85th percentile or higher.^13,14^ Our hypotheses were that increased risk perception and awareness would be associated with greater engagement in diabetes risk-reducing behaviors, whereas potential barriers would be associated with lower engagement (Figure).

**Figure.**
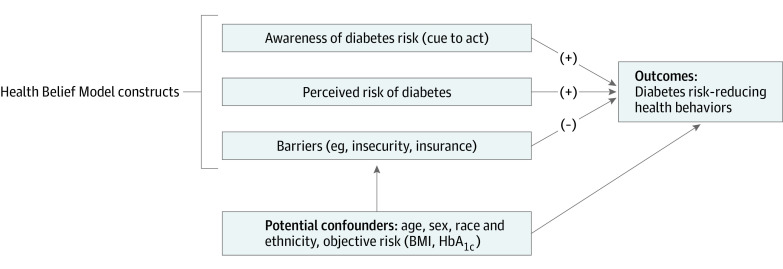
Conceptual Model Depicting Hypothesized Associations Between Health Belief Model Constructs and Diabetes Risk-Reducing Health Behaviors BMI indicates body mass index (calculated as weight in kilograms divided by height in meters squared); HbA_1c_, hemoglobin A_1c_.

## Methods

### Study Population

This cross-sectional study included youths aged 12 to 17 years of age with BMI in the 85th percentile or higher for age and sex and with available hemoglobin A_1c_ (HbA_1c_) measurement in the National Health and Nutrition Examination Survey (NHANES), waves 2011 to 2012, 2013 to 2014, 2015 to 2016, and 2017 to 2018. Patients with known diabetes and those who did not answer diabetes risk-related survey questions were excluded (eFigure in Supplement 1). NHANES, a large program conducted by the National Center for Health Statistics, uses physical examinations and interviews including demographic, socioeconomic, and health-related questions to evaluate disease prevalence and risk factors in a population selected to be representative of the US population after survey weighting. Race and ethnicity data were classified by self-report and categorized as Hispanic Mexican American, other Hispanic, and non-Hispanic Asian, Black, White, or other race, including American Indian or Alaskan Native, Native Hawaiian or Pacific Islander, other, or multiracial. Race and ethnicity were assessed due to the disproportionately higher incidence of type 2 diabetes among youth from minoritized racial and ethnic backgrounds. Informed consent was obtained from parents or legal guardians, and all survey cycles were approved by the National Center for Health Statistics Research Ethics Review Board. This study followed the Strengthening the Reporting of Observational Studies in Epidemiology (STROBE) reporting guideline.

We chose an age range of 12 to 17 years to maintain consistency in survey questions regarding diabetes risk and physical activity, which vary by age. All participants were invited to answer diabetes, nutrition, physical activity, and weight-related questionnaires. Physical activity and weight-related questionnaires were answered by participants directly. For nutrition and diabetes surveys, parent proxies answered for participants younger than 16 years. For this reason, differences in reported risk perception and awareness by age (younger than 16 years vs 16 years or older) were assessed; if significantly different, sensitivity analyses were conducted to evaluate for evidence of response bias. Survey question numbers, respondent type (parent or child) and phrasing are listed in the eTable in Supplement 1.

### Independent Variables: Health Belief Model Measures

#### Diabetes Risk Perception and Awareness

We used the Diabetes Questionnaire to assess risk perception and awareness. Risk perception was defined using the question, “Do you feel you could be at risk for diabetes or prediabetes?” while risk awareness was an affirmative to either “Have you ever been told by a doctor or other health professional that you have any of the following: prediabetes, impaired fasting glucose, impaired glucose tolerance, borderline diabetes or that your blood sugar is higher than normal but not high enough to be called diabetes or sugar diabetes?” (prediabetes) or “Have you ever been told by a doctor or other health professional that you have health conditions or a medical or family history that increases your risk for diabetes?” (diabetes risk).

#### Potential Barriers

To assess potential barriers to health behavior change, we included measures of household food security (full, marginal, low, very low), health insurance (public, private, other, none), health care access (whether/where/frequency routine medical care sought, mental health care utilization), income (annual household income and income to poverty ratio), and household size (total household members).

### Outcomes: Diabetes Risk-Reducing Health Behaviors

Measures included consumption of fast food, pizza, or non–home-prepared meals, physical activity and sedentary time, and frequency of attempted weight loss. For cycles 2011 to 2016, physical activity was assessed as total minutes spent in moderate or vigorous activity per week, determined using reported number of days per week spent in moderate and vigorous physical activity for recreation and for work, multiplied by the number of minutes spent in each activity on a typical active day. For cycle 2017 to 2018, physical activity was defined as the number of days per week with at least 60 minutes of moderate or vigorous physical activity. For all cycles, sedentary activity was evaluated using hours per day watching TV or using video games or computer for non-school activities. Additionally, for cycles 2011 to 2016, sedentary activity was also evaluated using minutes per day spent sitting.

### Potential Confounders

In addition to age, sex, and self-described race and ethnicity, we included measures of objective diabetes risk, including relative obesity (measured by BMI *z* score) and hyperglycemia (measured by HbA_1c_). The full NHANES sample (age ≥12 years) was eligible for measurement of HbA_1c_ (normal: <5.7%; prediabetes range: 5.7%-6.4%; diabetes range: ≥6.5%; to convert percentage of total hemoglobin to proportion of total hemoglobin, multiply by 0.01; to convert to millimoles per mole, multiply by 10.93 and subtract by 23.50). Due to the small subsample of participants in our age range and BMI range who completed fasting laboratory measurement, fasting glucose was not evaluated in this study.

### Statistical Analysis

We report demographic and clinical characteristics using summary statistics with 95% CIs. We used linear and logistic regression to evaluate the association between the Health Belief Model constructs and diabetes risk-reducing health behaviors. We created individual models for each behavioral outcome. We report physical activity outcomes and sedentary outcomes separately for cycles 2011 to 2016 and 2017 to 2018 because of the change in reporting, with the exception of screen time due to its consistency across all cycles. Due to collinearity of several barriers related to poverty, we used univariable regression to determine the subset for inclusion in multivariable models. We initially included barriers with *P* < .15 in univariable models, then used backward elimination, sequentially eliminating barrier variables with *P* > .05. The Health Belief Model constructs of risk perception and awareness were retained in all final models. Separate models are presented without and with potential confounders of age, sex, race and ethnicity, BMI *z* score, and HbA_1c_.

Analyses were performed using Stata 17 (StataCorp) from February 2022 to February 2023. All analyses incorporated appropriate sample weights^15^ and used a 2-sided α = .05 for statistical significance. We used Stata survey procedures to account for differences in response in the sample and the unequal probability of sample selection. To ensure appropriate point and variance estimates for the subsample used in our study, we used Stata’s *svy:subpop()* option. Missing data was not imputed. Relative standard errors were calculated to evaluate reliability of estimates; estimates with relative standard error greater than 30% (less reliable)^16^ are denoted.

## Results

### Cohort Characteristics

The cohort consisted of 1341 individuals representing 8 716 794 US youths aged 12 to 17 years with BMI in the 85th percentile or higher for age and sex. Mean age was 15.0 years (95% CI, 14.9-15.2 years), and the mean BMI *z* score was 1.76 (95% CI, 1.73-1.79). Table 1 demonstrates the demographic characteristics of the overall cohort, as well as by normal (<5.7%) vs elevated (≥5.7%) HbA_1c_. Elevated HbA_1c_ was present in approximately 9% of the cohort (prediabetes range HbA_1c_ [5.7%-6.4%]: 8.3% [95% CI, 6.5%-10.5%]; diabetes range HbA_1c_ [≥6.5%]: 0.3% [95% CI, 0.1%-0.7%]). Approximately one-third of youths with elevated HbA_1c_ reported feeling at risk for diabetes (risk perception), significantly more than youths with normal HbA_1c_ (30.1% [95% CI, 23.1%-38.1%] vs 19.6% [95% CI, 16.4%-23.3%]; *P* = .006). Risk perception was also significantly higher among those aged 16 to 17 years than among those aged 12 to 15 years (aged 16 to 17 years: 26.4% [95% CI, 21.8%-31.6%]; unweighted n = 448, weighted n = 2 932 359; aged 12 to 15 years: 17.5% [95% CI, 14.1%-21.6%]; unweighted n = 893, weighted n = 5 784 435; *P* = .002). Among those with elevated HbA_1c_, 22.1% (95% CI, 15.2%-30.9%) reported an awareness of “diabetes risk,” whereas 6.9% (95% CI, 3.6%-12.9%) reported a previous diagnosis with prediabetes. However, due to potential unreliability of the point estimate of prediabetes awareness (relative standard error >30%), we used a combined measure of awareness of either “prediabetes” or “diabetes risk,” which was present in 26.5% (95% CI, 20.0%-34.2%) of youth with elevated HbA_1c_. This combined awareness was significantly higher among youth with elevated HbA_1c_ than among those with normal HbA_1c_ (*P* = .001). Unlike risk perception, risk awareness did not differ between those aged 12 to 15 years and 16 to 17 years. Among the overall cohort, although awareness of prediabetes increased with more recent survey cycles (odds ratio [OR], 1.50 [95% CI, 1.05-2.15), perception of diabetes risk fell (OR, 0.78 [95% CI, 0.64-0.95]).

**Table 1.** Cohort Characteristics for the Full Cohort and by Normal or Elevated HbA_1c_

Characteristic	Unweighted No. (weighted %) [95% CI]		
	Full cohort (unweighted n = 1341; weighted n = 8 716 794)	HbA_1c_	
		Normal (<5.7%) (unweighted n = 1180; weighted n = 7 969 590)	Elevated (≥5.7%) (unweighted n = 161, weighted n = 747 204)
Age, mean (95% CI), y	15.0 (14.9-15.2)	15.0 (14.8-15.2)	15.1 (14.8-15.3)
BMI *z* score, mean (95% CI)	1.76 (1.73-1.79)	1.74 (1.71-1.77)	2.05 (1.96-2.13)
Female	677 (50.9) [47.3-54.4]	607 (51.6) [47.8-55.5]	70 (42.6) [33.5-52.2]
Race and ethnicity			
Non-Hispanic Asian	88 (2.8) [2.1-3.7]	75 (2.6) [2.0-3.5]	13 (4.8) [2.6-8.7]
Non-Hispanic Black	351 (14.4) [11.8-20.0]	271 (12.8) [9.7-16.6]	80 (44.5) [32.8-56.8]
Non-Hispanic White	327 (48.7) [42.4-55.0]	311 (51.6) [45.4-57.7]	16 (17.5) [9.6-29.9]
Non-Hispanic other race^a^	91 (6.1) [4.5-8.3]	82 (6.2) [4.5-8.6]	9 (4.9) [2.1-11.1]
Mexican American	338 (18.9) [15.1-23.3]	308 (18.7) [15.0-23.2]	30 (20.6) [11.4-34.3]
Other Hispanic^a^	146 (8.0) [6.3-10.1]	133 (8.0) [6.3-10.2]	13 (7.7) [4.2-13.6]^b^
Risk perception (feel at risk)	285 (20.5) [17.4-24.0]	240 (19.6) [16.4-23.3]	45 (30.1) [23.1-38.1]
Risk awareness			
Told they have prediabetes	40 (2.7) [1.7-4.0]	30 (2.3) [1.4-3.6]	10 (6.9) [3.6-12.9]^b^
Told they are at risk for diabetes	220 (15.8) [13.7-18.2]	185 (15.3) [13.1-17.7]	35 (22.1) [15.2-30.9]
Told they are at risk for diabetes or had prediabetes	235 (16.6) [14.4-19.0]	194 (15.6) [13.4-18.1]	41 (26.5) [20.0-34.2]

Abbreviations: BMI, body mass index (calculated as weight in kilograms divided by height in meters squared); HbA_1c_, hemoglobin HbA_1c_.

^a^ Other race includes American Indian or Alaskan Native, Native Hawaiian or Pacific Islander, other, or multiracial.

^b^ Point estimate may be less reliable due to relative standard error >30%.

### Association of Health Belief Model Constructs and Health Behaviors

#### Nutrition

In multivariable logistic regression unadjusted for potential confounders, risk perception and awareness were not associated with frequency of consumption of fast food, frozen meals, or non–home-prepared meals (Table 2). However, several potential barriers, including food insecurity, larger household size, and public insurance, arose as being associated with positive nutrition-related health behaviors. After adjustment for potential confounders, older age was associated with 30% to 40% higher odds of consuming at least 4 fast food meals or non–home-prepared meals in the past week, whereas female sex (vs male) was associated with 40% lower odds of consumption of at least 4 frozen meals or pizzas in the past 30 days. The potential barrier of larger households (≥5 members vs 1-2 members) remained associated with lower reported consumption of non–home-prepared meals (OR, 0.4 [95% CI, 0.2-0.7]).

**Table 2.** Multivariable Logistic Regression Models of Nutrition Behaviors, With and Without Adjustment for Confounders

Nutrition behavior	≥4 Fast food meals in past week (2011-2018); n = 1318 (weighted = 8 552 764)		≥4 Frozen meals/pizzas in past 30 d (2011-2018); n = 1337 (weighted = 8 691 513)		≥4 Non–home-prepared meals in past week (2011-2018); n = 1337 (weighted = 8 691 513)	
	OR (95% CI)	aOR (95% CI)^a^	OR (95% CI)	aOR (95% CI)^a^	OR (95% CI)	aOR (95% CI)^a^
Risk perception	1.7 (0.9-3.2)	1.5 (0.7-3.3)	0.8 (0.5-1.2)	0.8 (0.5-1.3)	1.3 (0.8-2.1)	1.2 (0.8-2.0)
Risk awareness	1.1 (0.6-2.0)	1.0 (0.6-1.7)	1.4 (0.9-2.3)	1.5 (0.9-2.4)	0.9 (0.6-1.4)	0.9 (0.6-1.3)
Barrier: food security (marginal and low/very low, vs full)	0.8 (0.5-1.5); 0.6 (0.4-0.97)	0.8 (0.4-1.4); 0.6 (0.4-0.9)	NA	NA	NA	NA
Barrier: insurance type (public, other, none vs private)	NA	NA	NA	NA	0.5 (0.3-0.9); 1.4 (0.7-3.0); 0.7 (0.4-1.5)	0.6 (0.4-0.9); 1.4 (0.7-2.7); 0.8 (0.4-1.7)
Barrier: household size (3-4, ≥5 vs 1-2)	NA	NA	NA	NA	0.7 (0.3-1.4); 0.4 (0.2-0.7)	0.7 (0.3-1.4); 0.4 (0.2-0.7)
Significant confounders from adjusted models	NA	Age: 1.4 (1.2-1.6)	NA	Female: 0.6 (0.4-0.8); Mexican American: 0.4 (0.2-0.6); other Hispanic: 0.5 (0.3-0.9); non-Hispanic Asian: 0.4 (0.2-0.7)	NA	Age: 1.3 (1.2-1.5)

Abbreviations: aOR, adjusted odds ratio; NA, not applicable; OR, odds ratio.

^a^ Adjusted models include age, sex (reference: male), race and ethnicity (reference: non-Hispanic White), body mass index *z* score, and hemoglobin A_1c_ category (≥5.7% vs <5.7% [reference]).

#### Physical Activity and Sedentary Behavior

In multivariable linear regression unadjusted for potential confounders, risk perception was associated with more screen time (β = 0.6, approximately 36 minutes per day; 95% CI, 0.1-1.0), mostly driven by TV watching (β = 0.4, approximately 24 minutes per day; 95% CI, 0.1-0.6) as well as fewer days in the past week with at least 60 minutes of physical activity (β = −1.5; 95% CI, −2.4 to −0.5) (Table 3). Risk awareness was not associated with any physical activity or sedentary behaviors. Potential barriers were associated with outcomes that were both protective (larger households reporting lower screen time) and harmful (fewer minutes in moderate or vigorous activity associated with food insecurity and public insurance; fewer days per week active associated with routine health care obtained through the emergency department). After adjustment for potential confounders, risk perception remained associated with increased TV watching (β = 0.3 hours per day; 95% CI, 0.2 to 0.5 hours per day) and 1 less day per week with at least 60 minutes physical activity (β = −1.2; 95% CI, −2.0 to −0.4). The potential barriers of larger households (≥5 members vs 1 or 2) and public insurance (vs private) remained associated with lower screen time (β = −1.1 hours per day; 95% CI, −2.0 to −0.3 hours per day) and approximately 20 fewer minutes per day of physical activity (β = −20.7 minutes per day; 95% CI, −35.5 to −5.8 minutes per day), respectively. Higher BMI *z*-score was associated with more screen time, including TV watching and computer or video games, and more time spent sitting (β = 39.3 minutes per day; 95% CI, 7.0-71.6 minutes per day). Female sex was associated with lower screen time (β = −0.4, approximately 24 minutes; 95% CI, −0.7 to −0.02) but less time spent in moderate or vigorous physical activity (β = −29.5 minutes per day; 95% CI, −44.4 to −14.7 minutes per day) and fewer days physically active for at least 60 minutes in the past week (β = −1.0 days per week; 95% CI, −1.7 to −0.3 days per week).

**Table 3.** Multivariable Linear Regression Models of Physical Activity Behaviors, With and Without Adjustment for Confounders

Health Belief Model Construct or Confounder	Sedentary time								Physical activity			
	Hours screen time/d (computer plus TV) in past 30 d (2011-2018); n = 1309 (weighted = 8 532 604)		Hours/d watching TV in past 30 d (2011-2018); n = 1308 (weighted = 8 524 519)		Hours/d using computer or video games (non-school) in past 30 d (2011-2018); n = 1308 (weighted = 8 529 390)		Minutes/d spent sitting (2011-2016 only); n = 995 (weighted = 8 568 328)		Minutes/d in moderate/vigorous activity (work and recreation) (2011-2016 only); n = 860 (weighted = 7 380 722)		Days in past week physically active ≥60 min (2017-2018 only); n = 268 (weighted = 7 164 980)	
	β (95% CI)	aß (95% CI)^a^	β (95% CI)	aß (95% CI)^a^	β (95% CI)	aß (95% CI)^a^	β (95% CI)	aß (95% CI)^a^	β (95% CI)	aß (95% CI)^a^	β (95% CI)	aß (95% CI)^a^
Risk perception	0.6 (0.1 to 1.0)	0.4 (−0.1 to 0.9)	0.4 (0.1 to 0.6)	0.3 (0.02 to 0.5)	0.2 (−0.1 to 0.6)	0.1 (−0.2 to 0.5)	14.8 (−24.0 to 53.5)	3.8 (−39.4 to 46.9)	−10.7 (−26.4 to 5.0)	−6.0 (−21.7 to 9.6)	−1.5 (−2.4 to −0.5)	−1.2 (−2.0 to −0.4)
Risk awareness	0.07 (−0.4 to 0.5)	−0.05 (−0.5 to 0.4)	0.03 (−0.3 to 0.4)	−0.02 (−0.3 to 0.3)	0.08 (−0.2 to 0.4)	0.01 (−0.3 to 0.3)	35.2 (−6.5 to 76.8)	29.9 (−10.9 to 70.8)	−4.9 (−24.0 to 14.2)	−4.1 (−22.5 to 14.3)	0.8 (−0.1 to 1.7)	0.8 (−0.2 to 1.7)
Barrier: food security (marginal and low/very low, vs full)	NA	NA	NA	NA	NA	NA	NA	NA	−20.3 (−37.0 to −3.7); 13.4 (−1.0 to 27.8)	−20.5 (−35.7 to −5.3); 11.4 (−2.9 to 25.7)	NA	NA
Barrier: insurance type (public, other, none vs private)	NA	NA	NA	NA	NA	NA	NA	NA	−21.8 (−40.9 to −2.6); −41.1 (−66.8 to −15.3); −17.1 (−47.0 to 12.8)	−20.7 (−35.5 to −5.8); −39.2 (−59.3 to −19.1); −13.5 (−40.6 to 13.6)	NA	NA
Barrier: household size (3-4, ≥5 vs 1-2)	−0.6 (−1.4 to 0.2); −1.3 (−2.1 to −0.4)	−0.5 (−1.3 to 0.3); −1.1 (−2.0 to −0.3)	−0.5 (−0.9 to −0.01); −0.7 (−1.2 to −0.3)	−0.4 (−0.9 to 0.03); −0.7 (−1.1 to −0.2)	NA	NA	NA	NA	NA	NA	NA	NA
Barrier: routine health care by ED (yes, vs no)	NA	NA	NA	NA	NA	NA	NA	NA	NA	NA	−2.4 (−3.6 to −1.3)	−2.7 (−4.0 to −1.4)
Significant confounders from adjusted models	NA	BMI *z*: 0.6 (0.2 to 0.9); Age: 0.1 (0.01,0.2); Female: −0.4 (−0.7 to −0.02)	NA	BMI *z*: 0.3 (0.06 to 0.5)	NA	BMI *z*: 0.3 (0.1 to 0.5); Age: 0.1 (0.05,0.2); Female: −0.3 (−0.6 to −0.07); non-Hispanic Asian: 0.5 (0.2 to 0.9)	NA	BMI *z*: 39.3 (7.0 to 71.6); Age: −8.2 (−15.3 to −1.1)	NA	Age: 12.8 (8.9 to 16.6); Female: −29.5 (−44.4 to −14.7); non-Hispanic Asian vs non-Hispanic White: −26.2 (−42.9 to −9.6)	NA	Female: −1.0 (−1.7 to −0.3)

Abbreviations: aß, adjusted β; β, unadjusted coefficient from linear regression; BMI, body mass index (calculated as weight in kilograms divided by height in meters squared); ED, emergency department; NA, not applicable.

^a^ Adjusted models include age, sex (reference: male), race and ethnicity (reference: non-Hispanic White), BMI *z* score, and hemoglobin A_1c_ category (≥5.7% vs <5.7% [reference]).

#### Weight Loss Attempts

In multivariable linear regression unadjusted for potential confounders, risk perception and awareness were not associated with frequency of attempted weight loss (sometimes or a lot vs never in the past year). No potential barriers were associated with reported weight loss attempts. Significant potential confounders included higher BMI *z* score, female sex, and Mexican American and non-Hispanic Asian youth (as compared with non-Hispanic White youth) (Table 4).

**Table 4.** Multivariable Logistic Regression Models of Reported Weight Loss Attempts, With and Without Adjustment for Confounders

	Weight loss attempts in past year: sometimes/a lot vs never (2011-2018), unweighted n = 893 (weighted n = 5 674 066)	
	OR (95% CI)	aOR (95% CI)^a^
Risk perception	2.3 (1.0-5.1)	1.4 (0.6-3.5)
Risk awareness	1.62 (0.95-2.78)	1.3 (0.7-2.4)
Significant confounders from adjusted model	NA	BMI *z*: 3.3 (1.7-6.3); female: 2.1 (1.2-3.5); age: 1.4 (1.3-1.6); Mexican American: 2.1 (1.1-3.9); non-Hispanic Asian: 4.8 (1.1-21.0)

Abbreviations: aOR, adjusted odds ratio; BMI, body mass index (calculated as weight in kilograms divided by height in meters squared); NA, not applicable; OR, odds ratio.

^a^ Adjusted models include age, sex (reference: male), race and ethnicity (reference: non-Hispanic White), BMI *z* score, and hemoglobin A_1c_ category (≥5.7% vs <5.7% [reference]).

### Sensitivity Analysis

Due to higher prevalence of risk perception among youths aged 16 to 17 years (self-report) vs youths aged 12 to 15 years (parent proxy), analyses were repeated in the subgroup aged 16 to 17 years (unweighted n = 448). Associations between time spent watching TV or days physically active and risk perception were no longer significant. Logistic regression to assess the association between attempted weight loss and risk perception or awareness was not possible because only 3 respondents in the subsample had not tried to lose weight in the past year, and all those who reported feeling at risk had attempted weight loss.

## Discussion

In this nationally representative sample of US youths at higher risk for type 2 diabetes due to elevated BMI, we found that risk perception and risk awareness were not associated with greater engagement in diabetes risk-reducing behaviors. Instead, greater diabetes risk perception was associated with more time spent watching TV and fewer days of adequate physical activity, whereas awareness was not associated with reported health behaviors. As posited by the Health Belief Model, some health care access barriers were associated with adverse patterns of health behaviors such as lower levels of physical activity. However, other potential barriers appeared protective, including larger household size, which was associated with less time spent watching TV and lower consumption of food prepared outside of the home.

Although our cohort included youth at higher risk for type 2 diabetes based on elevated BMI, perceived risk and awareness of diabetes risk was low overall. Even among youth with elevated HbA_1c_, who were at objectively higher risk for diabetes, only 30% reported feeling at risk, and 26.5% reported knowing they were at higher risk based on previous evaluation. Thus, nearly 75% of youth with elevated HbA_1c_ denied previous knowledge of their diabetes risk. Notably, our estimate of undiagnosed elevated HbA_1c_ consists nearly entirely of HbA_1c_ in the prediabetes range and should not be interpreted as undiagnosed diabetes, which was very rare, consistent with previous reports.^17^ However, our estimate of undiagnosed prediabetes is substantially higher than that reported in a recent systemic review that estimated the prevalence of undiagnosed prediabetes in adolescents aged 12 to 19 years globally to be 3.3% to 14.3%, although this estimate was affected by heterogeneity in definitions of prediabetes.^18^

Prediabetes-range HbA_1c_ can, at times, reflect only a transient worsening of insulin resistance during puberty and so may not carry the same clinical importance as a diagnosis of prediabetes in adults.^19^ However, elevated HbA_1c_ may also serve as a tangible marker of risk for some families, potentially leading to an interruption of ongoing rapid weight gain.^20^ We did find that, prior to adjustment for confounders, risk perception was associated with double the odds of attempted weight loss among adolescents, although this did not correspond to greater engagement in healthier diet or physical activity behaviors evaluated in our study. Notably, adjustment for BMI demonstrated that the association between risk perception and weight loss attempts was likely due to a reverse correlation: youth with higher BMI more frequently attempted to lose weight, and these youth may also have felt at greater risk for diabetes. This also likely explains the association between higher risk perception and both the reported lower amount of physical activity and the greater time spent watching TV. A prospective study is needed to definitively evaluate potential causal relationships between risk perception and health behaviors, as well as whether modification of risk perception could impact behaviors.

Notably, some presumed barriers to health behavior change in this study were unexpectedly associated with healthier behavioral patterns. Namely, lower reported screen time and less frequent consumption of food prepared outside of the home were reported for youth from larger households, and frequent consumption of fast food was less common among youth with household food insecurity. Although lower reported screen time is encouraging, overall time spent sedentary did not differ by household size. Similarly, although eating food prepared outside of the home was less common for larger households, this may not translate to better dietary quality. Other barriers, including health care access and food insecurity, were associated with lower reported physical activity and higher screen time. These findings suggest that traditional markers of economic disadvantage may have differential impacts on diabetes risk-reducing behaviors, whether nutrition- or activity-related.

Another important consideration for health behavior change in adolescents is risk awareness and perception of both adolescents and their caregivers. In our study, risk perception was assessed indirectly for youths 12 to 15 years old via a parent proxy, and directly for those aged 16 to 17 years. Our sensitivity analysis suggests that the association of this indirect measurement was minimal, but future studies may find even stronger associations between risk perception and health behaviors if adolescents are asked directly. Our finding that youth with lower rates of physical activity perceive themselves to be at higher risk of diabetes suggests that the failure to engage in healthy lifestyle behaviors is not a knowledge gap, but perhaps the result of limited self-efficacy^21^ or other barriers to health behavior change that were not measurable via NHANES. Further exploration of how risk perception emerges and its causal role in health behaviors may also inform future intervention design, as traditional motivational interviewing alone has not been demonstrated to be effective for treating adolescents with overweight and obesity.^22^

### Limitations

Our study has several strengths, including the large, nationally representative cohort spanning nearly a decade and the availability of comprehensive surveys, objective body size, and laboratory measures. We acknowledge limitations related to the observational, cross-sectional study design, including our inability to draw conclusions regarding causality between risk perception and health behaviors. Physical activity and nutrition assessment was based on self-report, rather than objective measurement. Laboratory measures were only obtained once, so persistence of dysglycemia or previously recognized dysglycemia is unknown. We did not include glucose in our definition of hyperglycemia, which may have limited our ability to detect objective risk as a driver of health behaviors. Birth history to assess diabetes risk factors of maternal gestational diabetes or small for gestational age birth weight was not available. We also did not assess additional risk factors such as polycystic ovary syndrome, presence of acanthosis nigricans, dyslipidemia, or hypertension, and documentation of family history of diabetes was too limited to assess reliably.^14^ The study’s duration included data from 2011 to 2018, over which time the incidence of prediabetes increased,^1^ which may have affected risk perception and awareness in youths and their families. Although we did find that awareness increased marginally, counterintuitively, risk perception decreased.

## Conclusions

In this nationally representative cross-sectional study, diabetes risk perception and awareness were not associated with healthier lifestyle, highlighting that raising risk awareness alone may be insufficient to motivate behavior changes. These findings suggest that further work is needed to develop evidence-based interventions that identify and reduce barriers to effective lifestyle change in youth.

## Notes

Supplement 1. **eTable.** Questions and Questionnaires Used, Target Age Range, and Respondent Detail **eFigure.** Consort Diagram

Supplement 2.
Data Sharing Statement
