# Supplementary material for: Perception and Awareness of Diabetes Risk and Reported Risk-Reducing Behaviors in Adolescents
Source: JAMA Netw Open. 2023 May 3;6(5):e2311466. doi: 10.1001/jamanetworkopen.2023.11466 (PMC10157422; doi:10.1001/jamanetworkopen.2023.11466)
Supplement: Supplement 1. — eTable. Questions and Questionnaires Used, Target Age Range, and Respondent Detail eFigure. Consort Diagram [file jamanetwopen-e2311466-s001.pdf]

Supplemental Online Content

Chu P, Patel A, Helgeson V, Goldschmidt AB, Ray MK, Vajravelu ME. Perception and awareness of diabetes risk and reported risk-reducing behaviors in adolescents. *JAMA Netw Open.* 2023;6(5):e2311466. doi:10.1001/jamanetworkopen.2023.11466

eTable. Questions and Questionnaires Used, Target Age Range, and Respondent Detail

eFigure. Consort Diagram

This supplemental material has been provided by the authors to give readers additional information about their work.

© 2023 Chu P et al. *JAMA Network Open.*

eTable. Questions and questionnaires used, target age range, and respondent detail					
**Question number**	**Questionnaire**	**Cycle**	**Topic**	**Age range**	**Respondent (Child = C, Parent = P)**
DIQ010	DIQ_J	2017-2018	Doctor told you have diabetes	0-150 years	C>=16y, P<16y
DIQ160	DIQ_J	2017-2018	Ever told you have prediabetes	12-150 years	C>=16y, P<16y
DIQ170	DIQ_J	2017-2018	Ever told you have health risk for diabetes	12-150 years	C>=16y, P<16y
DIQ172	DIQ_J	2017-2018	Feel at risk of diabetes	12-150 years	C>=16y, P<16y
DBD895	DBQ_J	2017-2018	Meals prepared not at home in past week	1-150 years	C>=16y, P<16y
DBD900	DBQ_J	2017-2018	Fast food or pizza in past week	1-150 years	C>=16y, P<16y
DBD910	DBQ_J	2017-2018	Frozen meals or pizza in past 30 days	1-150 years	C>=16y, P<16y
PAQ710	PAQY_J	2017-2018	Hours watch TV or videos for past 30 days	2-17 years	C
PAQ715	PAQY_J	2017-2018	Hours use computer last 30 days	2-17 years	C
PAQ706	PAQY_J	2017-2018	Days physically active >=60min	2-17 years	C
WHQ520	WHQMEC_J	2017-2018	How often tried to lose weight	8-15 years	C
WHQ070	WHQ_J	2017-2018	Tried to lose weight in past year	16-150 years	C
HIQ011	HIQ_J	2017-2018	Covered by health insurance	0-150 years	C>=16y, P<16y
HIQ031A	HIQ_J	2017-2018	Covered by private insurance	0-150 years	C>=16y, P<16y
HIQ031F	HIQ_J	2017-2018	Covered by military insurance	0-150 years	C>=16y, P<16y
HIQ031D	HIQ_J	2017-2018	Covered by Medicaid	0-150 years	C>=16y, P<16y
HIQ031E	HIQ_J	2017-2018	Covered by CHIP	0-150 years	C>=16y, P<16y
HIQ031H	HIQ_J	2017-2018	Covered by state-sponsored health plan	0-150 years	C>=16y, P<16y

© 2023 Chu P et al. *JAMA Network Open.*

HIQ031I	HIQ_J	2017-2018	Covered by other government insurance	0-150 years	C>=16y, P<16y
IND235	INQ_J	2017-2018	Monthly family income	0-150 years	P
INDFMMPI	INQ_J	2017-2018	Family monthly poverty level index	0-150 years	P
INDFMMPC	INQ_J	2017-2018	Family monthly poverty level category	0-150 years	P
DIQ010	DIQ_I	2015-2016	Doctor told you have diabetes	0-150 years	C>=16y, P<16y
DIQ160	DIQ_I	2015-2016	Ever told you have prediabetes	12-150 years	C>=16y, P<16y
DIQ170	DIQ_J	2015-2016	Ever told you have health risk for diabetes	12-150 years	C>=16y, P<16y
DIQ172	DIQ_J	2015-2016	Feel at risk of diabetes	12-150 years	C>=16y, P<16y
DBD895	DBQ_I	2015-2016	Meals prepared not at home in past week	1-150 years	C>=16y, P<16y
DBD900	DBQ_I	2015-2016	Fast food or pizza in past week	1-150 years	C>=16y, P<16y
DBD910	DBQ_J	2015-2016	Frozen meals or pizza in past 30 days	1-150 years	C>=16y, P<16y
PAQ655	PAQ_I	2015-2016	Days/week engaged in vigorous sports, fitness, or recreational activity	12-150 years	C
PAD660	PAQ-I	2015-2016	Minutes spent in vigorous sports, fitness, or recreational activity on typical day	12-150 years	C
PAQ670	PAQ-I	2015-2016	Days/week engaged in moderate sports, fitness, or recreational activity	12-150 years	C
PAD675	PAQ-I	2015-2016	Minutes spent in moderate sports, fitness, or recreational activity on typical day	12-150 years	C
PAD680	PAQ-I	2015-2016	Minutes spent sitting on typical day	12-150 years	C

© 2023 Chu P et al. *JAMA Network Open.*

PAQ710	PAQ_I	2015-2016	Hours watch TV or videos for past 30 days	2-17 years	C
PAQ715	PAQ_I	2015-2016	Hours use computer last 30 days	2-17 years	C
WHQ520	WHQMEC_I	2015-2016	How often tried to lose weight	8-15 years	C
WHQ070	WHQ_I	2015-2016	Tried to lose weight in past year	16-150 years	C
HIQ011	HIQ_I	2015-2016	Covered by health insurance	0-150 years	C>=16y, P<16y
HIQ031A	HIQ_I	2015-2016	Covered by private insurance	0-150 years	C>=16y, P<16y
HIQ031F	HIQ_I	2015-2016	Covered by military insurance	0-150 years	C>=16y, P<16y
HIQ031D	HIQ_I	2015-2016	Covered by Medicaid	0-150 years	C>=16y, P<16y
HIQ031E	HIQ_I	2015-2016	Covered by CHIP	0-150 years	C>=16y, P<16y
HIQ031H	HIQ_I	2015-2016	Covered by state-sponsored health plan	0-150 years	C>=16y, P<16y
HIQ031I	HIQ_I	2015-2016	Covered by other government insurance	0-150 years	C>=16y, P<16y
IND235	INQ_I	2015-2016	Monthly family income	0-150 years	P
INDFMMPI	INQ_I	2015-2016	Family monthly poverty level index	0-150 years	P
INDFMMPC	INQ_I	2015-2016	Family monthly poverty level category	0-150 years	P
DIQ010	DIQ_H	2013-2014	Doctor told you have diabetes	0-150 years	C>=16y, P<16y
DIQ160	DIQ_H	2013-2014	Ever told you have prediabetes	12-150 years	C>=16y, P<16y
DIQ170	DIQ_H	2013-2014	Ever told you have health risk for diabetes	12-150 years	C>=16y, P<16y
DIQ172	DIQ_H	2013-2014	Feel at risk of diabetes	12-150 years	C>=16y, P<16y
DBD895	DBQ_H	2013-2014	Meals prepared not at home in past week	1-150 years	C>=16y, P<16y

© 2023 Chu P et al. *JAMA Network Open.*

DBD900	DBQ_H	2013-2014	Fast food or pizza in past week	1-150 years	C>=16y, P<16y
DBD910	DBQ_H	2013-2014	Frozen meals or pizza in past 30 days	1-150 years	C>=16y, P<16y
PAQ655	PAQ_H	2013-2014	Days/week engaged in vigorous sports, fitness, or recreational activity	12-150 years	C
PAD660	PAQ_H	2013-2014	Minutes spent in vigorous sports, fitness, or recreational activity on typical day	12-150 years	C
PAQ670	PAQ_H	2013-2014	Days/week engaged in moderate sports, fitness, or recreational activity	12-150 years	C
PAD675	PAQ_H	2013-2014	Minutes spent in moderate sports, fitness, or recreational activity on typical day	12-150 years	C
PAD680	PAQ_H	2013-2014	Minutes spent sitting on typical day	12-150 years	C
PAQ710	PAQ_H	2013-2014	Hours watch TV or videos for past 30 days	2-17 years	C
PAQ715	PAQ_H	2013-2014	Hours use computer last 30 days	2-17 years	C
WHQ520	WHQMEC_H	2013-2014	How often tried to lose weight	8-15 years	C
WHQ070	WHQ_H	2013-2014	Tried to lose weight in past year	16-150 years	C
HIQ011	HIQ_H	2013-2014	Covered by health insurance	0-150 years	C>=16y, P<16y
HIQ031A	HIQ_H	2013-2014	Covered by private insurance	0-150 years	C>=16y, P<16y
HIQ031F	HIQ_H	2013-2014	Covered by military insurance	0-150 years	C>=16y, P<16y
HIQ031D	HIQ_H	2013-2014	Covered by Medicaid	0-150 years	C>=16y, P<16y
HIQ031E	HIQ_H	2013-2014	Covered by CHIP	0-150 years	C>=16y, P<16y
HIQ031H	HIQ_H	2013-2014	Covered by state-sponsored health plan	0-150 years	C>=16y, P<16y
HIQ031I	HIQ_H	2013-2014	Covered by other government insurance	0-150 years	C>=16y, P<16y

© 2023 Chu P et al. *JAMA Network Open.*

IND235	INQ_H	2013-2014	Monthly family income	0-150 years	P
INDFMMPI	INQ_H	2013-2014	Family monthly poverty level index	0-150 years	P
INDFMMPC	INQ_H	2013-2014	Family monthly poverty level category	0-150 years	P
DIQ010	DIQ_G	2011-2012	Doctor told you have diabetes	0-150 years	C>=16y, P<16y
DIQ160	DIQ_G	2011-2012	Ever told you have prediabetes	12-150 years	C>=16y, P<16y
DIQ170	DIQ_G	2011-2012	Ever told you have health risk for diabetes	12-150 years	C>=16y, P<16y
DIQ172	DIQ_G	2011-2012	Feel at risk of diabetes	12-150 years	C>=16y, P<16y
DBD895	DBQ_G	2011-2012	Meals prepared not at home in past week	1-150 years	C>=16y, P<16y
DBD900	DBQ_G	2011-2012	Fast food or pizza in past week	1-150 years	C>=16y, P<16y
DBD910	DBQ_G	2011-2012	Frozen meals or pizza in past 30 days	1-150 years	C>=16y, P<16y
PAQ655	PAQ_G	2011-2012	Days/week engaged in vigorous sports, fitness, or recreational activity	12-150 years	C
PAD660	PAQ_G	2011-2012	Minutes spent in vigorous sports, fitness, or recreational activity on typical day	12-150 years	C
PAQ670	PAQ_G	2011-2012	Days/week engaged in moderate sports, fitness, or recreational activity	12-150 years	C
PAD675	PAQ_G	2011-2012	Minutes spent in moderate sports, fitness, or recreational activity on typical day	12-150 years	C
PAD680	PAQ_G	2011-2012	Minutes spent sitting on typical day	12-150 years	C
PAQ710	PAQ_G	2011-2012	Hours watch TV or videos for past 30 days	2-17 years	C
PAQ715	PAQ_G	2011-2012	Hours use computer last 30 days	2-17 years	C
WHQ520	WHQMEC_G	2011-2012	How often tried to lose weight	8-15 years	C

© 2023 Chu P et al. *JAMA Network Open.*

WHQ070	WHQ_G	2011-2012	Tried to lose weight in past year	16-150 years	C
HIQ011	HIQ_G	2011-2012	Covered by health insurance	0-150 years	C>=16y, P<16y
HIQ031A	HIQ_G	2011-2012	Covered by private insurance	0-150 years	C>=16y, P<16y
HIQ031F	HIQ_G	2011-2012	Covered by military insurance	0-150 years	C>=16y, P<16y
HIQ031D	HIQ_G	2011-2012	Covered by Medicaid	0-150 years	C>=16y, P<16y
HIQ031E	HIQ_G	2011-2012	Covered by CHIP	0-150 years	C>=16y, P<16y
HIQ031H	HIQ_G	2011-2012	Covered by state-sponsored health plan	0-150 years	C>=16y, P<16y
HIQ031I	HIQ_G	2011-2012	Covered by other government insurance	0-150 years	C>=16y, P<16y
IND235	INQ_G	2011-2012	Monthly family income	0-150 years	P
INDFMMPI	INQ_G	2011-2012	Family monthly poverty level index	0-150 years	P
INDFMMPC	INQ_G	2011-2012	Family monthly poverty level category	0-150 years	P

© 2023 Chu P et al. *JAMA Network Open.*

eFigure. Consort Diagram

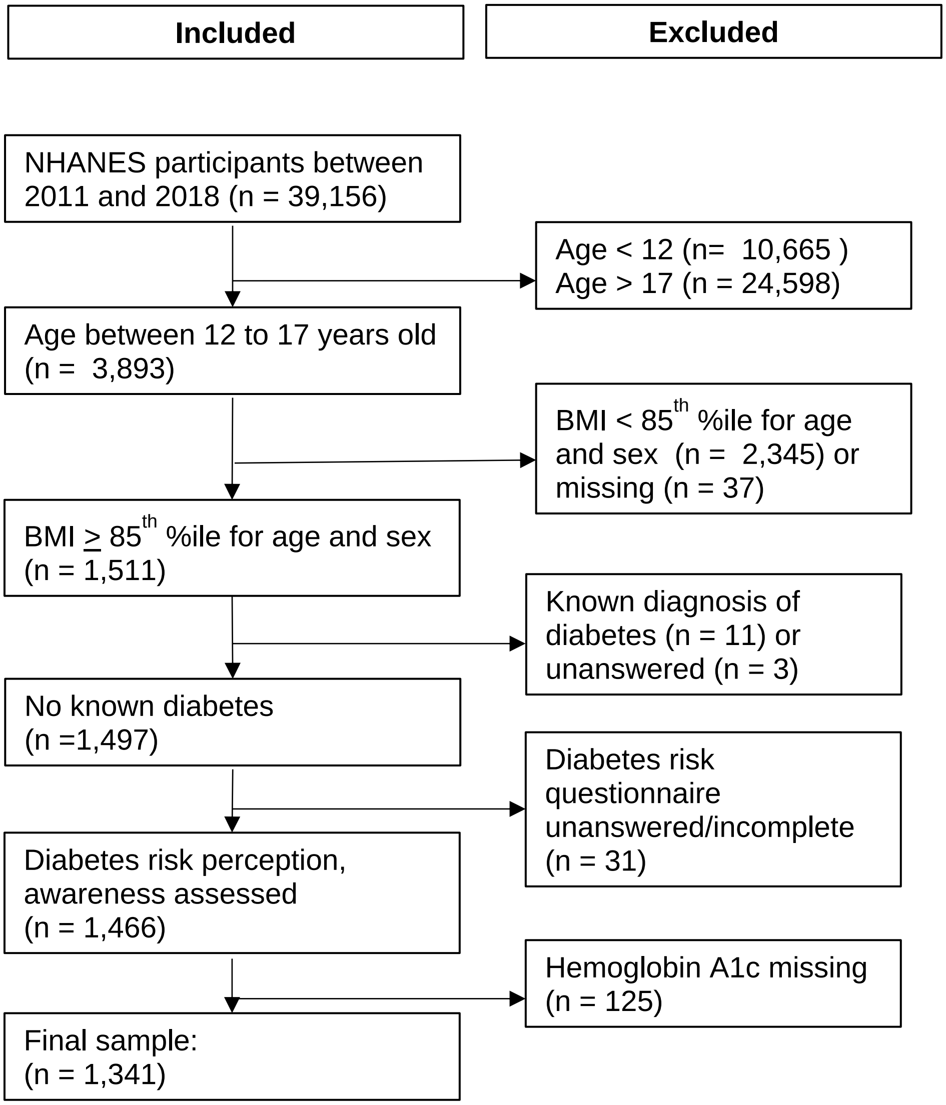

© 2023 Chu P et al. *JAMA Network Open.*
