# Supplementary material for: Perception and Awareness of Diabetes Risk and Reported Risk-Reducing Behaviors in Adolescents
Source: JAMA Netw Open. 2023 May 3;6(5):e2311466. doi: 10.1001/jamanetworkopen.2023.11466 (PMC10157422; doi:10.1001/jamanetworkopen.2023.11466)
Supplement: Supplement 2. — Data Sharing Statement [file jamanetwopen-e2311466-s002.pdf]

Data Sharing Statement

Chu. Perception and Awareness of Diabetes Risk and Reported Risk-Reducing Behaviors in Adolescents. *JAMA Netw Open*. Published May 03, 2023.
doi:10.1001/jamanetworkopen.2023.11466

Data
Data available: No

Additional Information
Explanation for why data not available: The NHANES database, which contains deidentified participant data, can be freely accessed and downloaded at any time at: https://wwwn.cdc.gov/nchs/nhanes/Default.aspx.
